# The Orthographic Ambiguity of the Arabic Graphic System: Evidence from a Case of Central Agraphia Affecting the Two Routes of Spelling

**DOI:** 10.1155/2022/8078607

**Published:** 2022-11-16

**Authors:** Assia Boumaraf, Sonia Bekal, Joël Macoir

**Affiliations:** ^1^Centre de Recherche Scientifique et Technique pour le Développement de la Langue Arabe, Alger, Algeria; ^2^Faculté de Médecine, Département de Réadaptation, Université Laval, Québec (QC), Canada; ^3^Centre de Recherche CERVO-CERVO Brain Research Center, Québec (QC), Canada

## Abstract

The Arabic writing system includes ambiguities that create difficulties in spelling. These ambiguities relate mainly to the long vowels, some phoneme-grapheme conversions, lexical particularities, and the connectivity of letters. In this article, the first to specifically explore acquired spelling impairments in an Arabic-speaking individual, we report the case of CHS, who presented with agraphia following a stroke. Initial testing indicated substantial impairment of CHS's spelling abilities in the form of mixed agraphia. The experimental study was specifically designed to explore the influence of the orthographic ambiguity of the Arabic graphemic system on CHS's spelling performance. The results revealed that CHS had substantial difficulties with orthographic ambiguity and tended to omit ambiguous graphemes. Some of the errors she produced suggested reliance on the sublexical route of spelling, while others rather reflected the adoption of the lexical-semantic route. These findings from a case involving a non-Western, non-Indo-European language contribute to discussions of theoretical models of spelling. They show that CHS's pattern of impairment is consistent with the *summation hypothesis*, according to which the lexical-semantic and the sublexical routes interactively contribute to spelling.

## 1. Introduction

The impairment of written language abilities is a frequent aftereffect of neurological disorders. However, although there are many studies on acquired dyslexia, acquired agraphia has been largely understudied. Most knowledge comes from single-case studies of individuals with agraphia resulting from stroke or neurodegenerative disease [1]. These studies have contributed to the development of cognitive neuropsychological models of the functional architecture of the spelling system. The dual-route model [2, 3] is one of the most influential. According to this model, written words are retrieved and produced through the activation of specialized, interconnected components that are implemented via two routes: the lexical-semantic route, which is used to spell familiar words, and the sublexical route, which is used to spell novel words or nonwords [4]. In the lexical-semantic route, which is involved in spelling-to-dictation or written naming, the concept corresponding to the object or idea to be expressed is first activated at the conceptual-semantic level. Then, this nonverbal conceptual representation is mapped onto a representation in the orthographic lexicon. Finally, the retrieved orthographic word form is placed in orthographic short-term memory, where it remains activated until it is converted into an appropriate format (i.e., spelling aloud, typing, or writing). In the sublexical route, which is mainly involved in spelling-to-dictation, the phonological input is serially transformed into a string of graphemes via phoneme-to-grapheme rules [5].

Arabic script differs from Roman script in many aspects. A concise description of the Arabic graphic system is therefore presented in the next section.

Arabic is written from right to left. Like other Semitic languages, its alphabet is mainly consonantal; it consists of 28 letters, which most of them are consonants. Thirteen letters have dots above or below the main symbol. Some letters have a single dot, such as بـ/b/ and نـ/n/, while other letters have two (ي/y/) or three dots (ش/š/).

The Arabic writing system is relatively transparent. The pronunciation of the letters does not change, regardless of the position they occupy, except for the two semivowels “w” and “y,” which can function as consonants (e.g., وردة/warda/ (rose) and يد/yad/ (hand)) or long vowels (e.g., u: →و as in /سوقsu:q/ (market) and i:→ as فيلin/fi:l/ (elephant)).

There are two types of vowels in Arabic: three short vowels (a, u, and i) and three long vowels (a:, u:, and i:). Short vowels are much more frequent than long vowels and are usually not written. The long vowels are represented by extended letters that denote the extension of the sound of the short vowels (e.g., با→ba: بو→bu: بي→bi:). In Arabic, the application of the phoneme-to-grapheme conversion rules generates consonants only. For example, the phonological form /kataba/ (he wrote) contained three consonants and three vowels a-a-a, while its orthographic form كتب is represented only by three consonants k, t, and b. The phonological form /kita:bu/ (book) contains the same consonants but one of its three vowels is long. Therefore, the corresponding orthographic form كتابُ is represented by four letters: three for the consonants and one for the long vowel. The fact that some vowels are represented by letters while others are not is one of the challenges in Arabic spelling.

Consonants and vowels are also distinguished by their morphological status: consonants generally refer to the root that conveys the word's main meaning (the substance), while vowels represent the word pattern (the mold) into which the consonants of the root are integrated to form words. For example, the words “writing,” “writer,” “library,” “is written,” and “books” share the same root (k, t, b) related to the notion of writing, expressed with vowels to form different words: /kita:ba/كتابة (writing), /ka:teb/كاتب (writer), /maktaba/مكتبة (library), /maktu:b/مكتوب (written), and /kutub/كُتُب (books). Therefore, the generation of words of the same family (having the same root) is governed by changes of the word pattern, and each morphological change is followed by a vocalic and subsequently by a graphic change.

The Arabic language is characterized by diglossia, a term used to describe a situation in which two types of language exist side by side in the community. These two types are Modern Standard Arabic and dialectal Arabic. Both are linguistically related, having the same phonological and lexical structures, but significantly different from each other in some respects. For example, compared to Modern Standard Arabic, dialectal Arabic is characterized by loss of some short vowels and interdental phonemes, loss of double forms, and loss of grammatical marks [6].

As defined by Beauvois and Dérouesné [7], an ambiguous phoneme is a phoneme that can be written in several different ways through the application of phoneme-grapheme conversion rules. Some phonemes are transcribed in different ways, depending on their sequential position in the word. In French for instance, the phoneme /s/ which is consistently written S (e.g., savon (soap)) might be written C before the letters E and I (e.g., citron (lemon)). In Arabic, each phoneme corresponds a particular grapheme. For example, the phoneme /b/ is always represented by the letter ب. There are however some few exceptions and sources of graphemic ambiguity. The long vowel a: can be written with two allographs, namely, ا and ى: the allograph ا can take the initial, middle, or final position in the word, while the allograph ى can only be expressed in the final position.

The Hamza is the only Arabic letter whose shape is determined not only by its position in the word in relation to other letters but also by phonetic aspects of the word. For this reason, Parkinson [8] considered the Hamza as the most variable aspect of the Arabic orthographic system. In fact, the Hamza can be represented by seven different allographs (ء ، ئـ ، ئ ، ا ، ؤ، أ ، آ), five of which consist of two letters; the Hamza ء and the letter base; the Hamza is placed above (i.e., the seat) (e.g., ء + ا = أ, ء + و = ؤ, ء+ ى = ئ). These seats are versions of the three long Arabic vowels a:, u:, and i: named, respectively, alif, waaw, and yaa. It is the choice of the seat itself, or lack thereof, that is the source of the observed variable usage [8]. The choice of allograph is governed by contextual rules that can be classified as positional or vocalic environment rules, that is, the vowels that precede and follow the Hamza [9]. These rules are complex and difficult to apply and, moreover, there are some exceptions. This complexity is for example demonstrated by the difficulties experienced by children in writing the Hamza compared to the other Arabic letters [10].

In addition to graphemic ambiguity, Arabic script is characterized by lexical ambiguity. In Arabic, lexical ambiguity is determined by the presence or absence of long vowels and the presence or absence of the ta marbu:ṭa, a final letter often used to mark nouns and adjectives as feminine. For example, a (male) teacher is معلم/muʕallim/, while a (female) teacher is spelled by adding a ta marbu:ṭa معلمة/muʕallimah/. However, the case ending of the feminine form must be omitted with the preservation of the vowel /a/, and the word is pronounced /muʕalima/. This adds a significant degree of ambiguity to written forms, since the speller must know when to write a ta marbu:ṭa at the end of a word, even though it is not pronounced.

Finally, another characteristic of Arabic orthography is that letters can be connected to each other, and they have different shapes depending on their position in a word. This means that complete Arabic words have specific shapes that generally differ from those of other words, even those that use the same letters in different arrangements. For example, the two words سعد/saʕd/ (prosperity) and عدس/ʕadas/ (lentil) share the same three letters س ع د (s, ʕ, d); although, the shapes of these words are distinctly different. Therefore, it is common for Arabic spellers to first write the global shape of a word and then add the dots above or below letters. In Arabic, word shape has been shown to influence reading performance in individuals with acquired deep dyslexia [11].

The effect of orthographic ambiguity in Arabic has not been overtly explored, but a few studies have examined childhood spelling difficulties of Arabic speakers [10, 12] and spellings errors made by foreign learners [13]. These studies reveal that the errors made by beginner learners are generally similar to those made by foreign learners. These errors consist mainly of phoneme-grapheme conversion errors, difficulty distinguishing letters with visually similar shapes, confusion between short and long vowels, difficulty applying context-sensitive rules for writing the Hamza ء and the grapheme t ت, and errors writing the ta marbu:ṭa. All these types of errors point to the use of the sublexical route of spelling; skillful spellers have acquired orthographic lexical knowledge that prevents such errors.

Most existing studies on acquired agraphia involve Western Indo-European languages such as English, French, or German, which mostly have deep orthography. A very few studies have been conducted on languages with shallow orthography, such as Italian (e.g., Luzzi, & al. [14]) and Spanish (e.g., Iribarren, Jarema & Lecours [15]). For example, Iribarren et al. [15] reported two cases of agraphia in Spanish, one of surface agraphia, and another of phonological agraphia. There are very few studies specifically exploring acquired agraphia in Arabic-speaking individuals with poststroke aphasia, and, more generally, there are very few studies dedicated to acquired agraphia in Semitic languages. El Alaoui Faris et al. [16] reported the case of an Arabic-speaking patient who suffered from a left-brain abscess, resulting in agraphia due to graphemic buffer disorder. In this patient, there was a clear effect of word length with a predominance of substitutions errors in the middle of the words. The errors were also mainly produced on vowels compared to consonants in different writing tasks such as writing to dictation and written picture naming. A similar acquired agraphia was also described in a Hebrew speaking patient [17]. More recently, Aldera and Balasubramanian [18] reported the presence of typical symptoms of central agraphia (i.e., phonological agraphia, graphemic buffer agraphia, and mixed agraphia) in a case series of 15 individuals with poststroke aphasia.

In this study, we tested the two-route model of spelling in CHS, an Arabic-speaking individual who presents with nonfluent aphasia and deep dyslexia associated with letter-by-letter reading. In CHS, we have showed that the reading impairment is due to decreased access to orthographic lexical representations [19]. Written production is also impaired in CHS. We assessed her spelling abilities to determine the functional origin of the deficit and then explored the impact of orthographic ambiguity on her writing skills.

## 2. Case Study

CHS is a 48-year-old Arabic-speaking woman who completed 17 years of education. She suffered a left-hemispheric ischemic stroke in August 2009, resulting in nonfluent aphasia and right hemiplegia. CHS is left-handed; so, the left hemispheric stroke did not affect the use of her dominant hand. Before her accident, CHS spoke three languages (Arabic, French, and English) and worked as a journalist. She spoke Algerian dialect and other Arabic dialects, but she usually used Modern Standard Arabic for her journalistic reports on Arabic channels. In everyday life, she usually used the Algerian Arabic dialect. CHS received rehabilitation services in physiotherapy and speech-language pathology during a three-month hospital stay in France. In March 2010, an encephalic MRI revealed lesions spanning the peri-Sylvian region of the left hemisphere as well as an ipsilateral occipital lesion localized in the posterior cerebral artery territory suggesting embolic stroke. After her return to Algeria, she refused to be assessed in another language than Arabic. Consequently, CHS's spelling assessment was conducted only in Arabic ten years' postonset.

CHS was assessed in Modern Standard Arabic with a battery of neuropsychological and language tests. Her performance on neuropsychological tests was compared against existing norms (see Table 1). Her performance on the language tests and on the experimental tasks (see experimental study) was compared to the results of five, age-, and education-matched controls (mean age = 46.6 years; mean education = 17 years), using modified *t*-tests [20]. As recommended by Crawford, Garthwaite, and Porter [20], an index of effect size was also calculated using CHS's score (*x*) and the mean ($\overset{®}{X}$) and standard deviation (SD) of the control sample. This index, denoted ZCC, is computed using the formula (*Zcc* = *X* − *X̅*/SD). For cases with no standard deviation for the controls (i.e., the control participants obtained perfect scores), CHS's scores were compared to those of the controls using chi-squared tests.

CHS and the control participants provided written informed consent prior to their participation in the study, in accordance with the Declaration of Helsinki (BMJ, 1991; 302 : 1194). This study was approved by the research ethics board at the Center of Scientific and Technical Research for the Development of Arabic Language of Algiers (CSTRDAL ## 422).

This assessment revealed that CHS had constructional apraxia, impaired verbal short-term and verbal working memory, impaired executive functions of mental flexibility and verbal inhibition, and impaired visuoperceptual abilities affecting the perceptual and associative steps of object recognition.

With respect to language, CHS showed impaired access to orthographic representations in the input lexicon and difficulty finding words in spoken production (see Table 2). With respect to phonological abilities, CHS showed mild impairment in the auditory discrimination task (i.e., judging if two stimuli are identical or not) as well as in the auditory lexical decision in which she had difficulty rejecting non-words (see Table 2). Her repetition abilities were perfectly preserved for words, while she had substantial difficulties for nonwords for which she mainly produced lexicalizations. CHS reading profile met most of the criteria for deep dyslexia, with the exception that her reading performance was not influenced by imageability, and with the presence of LBL reading, which is not common in this syndrome. An extensive description of the assessment of CHS's reading abilities was presented in a previous study [19].

CHS's spelling abilities were assessed using a battery of tasks, which are all developed in-house by the authors and all administered without a time limit.

CHS was first asked to copy in cursive attached letters: a set of 24 words presented with separated letters (e.g., عنب→ع ن ب) and then to perform the reverse operation (e.g., ع ن ب→عنب) without seeing the initial stimuli. Her performance was largely preserved (performance of control participants was flawless) on both directions of copying (separated to attached letters = 91.66%; attached to separated letters = 95.83%). The few errors she produced were all made on the Hamza. CHS's written and oral spelling abilities were compared with a writing to dictation task of 72 words (containing 3-5 letters and 1-3 syllables) administered in the two modalities in two separate sessions. As shown in Table 3, her performance was equally impaired in the two conditions of the task, excluding the presence of peripheral disorder of spelling.

CHS's performance was impaired in the written description of the bank robbery scene taken from the *Batterie Montréal-Toulouse d'examen linguistique de l'aphasie* [21] (see verbatim transcription in Appendix A). Her written production was characterized by difficulty finding words, omission of conjunctions, and spelling errors. Most of the errors consisted in nonphonological plausible and morphological errors.

As shown in Table 3, CHS's performance on writing letters dictated in a random order did not differ from that of the control participants. However, compared to the control participants, her performance was poor when asked to write syllables with and without long vowels.

CHS was also asked to write lists of words to dictation. As shown in Table 3, she exhibited impaired performance for high- and low-frequency words, high- and low-imageability words, and closed-class (conjunctions, prepositions, and pronouns) and open-class (verbs and nouns) words. Most of her errors consisted in nonphonological plausible errors (e.g., زهرة/zahra/ (flower)→دهروة/dahrawa/ (nonword)) and substitutions of visually similar letters (e.g., دخان/duḫa:n/ (smoke)→دجان/dudʒa:n/), as well as, in a lesser proportion, semantic substitutions, (e.g., جامع/dʒa:meʕ/(great mosque)→/مسجدmasdʒid/ (mosque), and morphological errors (e.g., /مكتبmaktab/ (desk)→مكتبة/maktaba/ (library). Examples of error types produced by CHS in spelling to dictation are presented in Appendix B.

CHS's performance was also substantially impaired in the nonword writing-to-dictation task, in which she mainly produced lexicalizations and nonphonologically plausible errors.

Finally, compared to control participants, CHS's performance was significantly impaired in the written picture naming test (see Table 3). She could not produce any response in 10 out of the 32 errors (31%) or produced errors on assigning dots but preserving the whole word form (6/32, 19%), semantic errors (4/32, 12.5%), omissions of long vowels (7/32, 22%), and of ta marbu:ṭa (4/32, 12.5%) as well as one morphological error (3%).

With respect to written abilities, the results of the background testing showed that CHS was substantially impaired in all tasks. She did not present with the characteristics of a peripheral writing disorder. Her spelling profile corresponded to a severe mixed agraphia affecting the written production of words and nonwords thus demonstrating impairment of both the lexical and the phonological routes.

The experimental study directly explored the influence of the orthographic ambiguity of the Arabic graphemic system on CHS's spelling performance.

## 3. Experimental Study: The Influence of Orthographic Ambiguity on Spelling

### 3.1. Methods

To explore the effect, if any, of orthographic ambiguity on CHS's spelling abilities, we designed two writing-to-dictation tasks. All the stimuli were derived from Modern Standard Arabic. The very large proportion of them exists in both Modern Standard Arabic and Algerian Arabic to avoid interference between the two registers. The first task comprised of four lists of 50 mono- and polysyllabic words with varying degrees of orthographic ambiguity: (1) unambiguous words (e.g., عسل/ʕasal/ (honey)), (2) ambiguous words containing one orthographic ambiguity (e.g., a long vowel: حساب/hisa:b/ (account)), (3) ambiguous words containing two orthographic ambiguities (e.g., a long vowel and a Hamza: تفاؤل/tafa:ʔul/ (optimism)), and (4) very ambiguous words containing three orthographic ambiguities (e.g., a long vowel, a Hamza, and a ta marbu:ṭa تأشيرة/taʔši:ra/ (visa)). All words were nouns, and the four lists were matched for lexical frequency according to the Aralex database [22]. Words were not controlled for letter length, a parameter not totally independent of orthographic ambiguity [7], especially in Arabic, where short vowels are not represented by letters. Words contained 1-4 syllables and 3-7 letters, 3-4 of which represented to the root and the others depicted the long vowels and/or the ta: marbu:ṭa relating to the word pattern. Simple words, by their nature, cannot not support long vowels, Hamza and ta: marbu:ṭa, so that they were made of 3 and 4 letters only. A list of 20 nonambiguous and 20 ambiguous nonwords, respecting orthotactic and phonotactic rules, was created and used for the third writing-to-dictation task. Only nonwords that CHS was able to correctly repeat were selected for this list. These nonwords, comprising 3-5 letters and 1-3 syllables, were created based on real, high-imageability Arabic words. The 200 words and the 40 nonwords were presented separately in random order to CHS and the control participants in writing-to-dictation tasks at several testing sessions. Each item was dictated and repeated if necessary, and participants could take as much time as needed to produce a response. One point was given to each correct response. When CHS or the control participants made an error and tried to correct it, the second response only was rated.

### 3.2. Results

As shown in Table 4, CHS's performance on the spelling-to-dictation task was severely impaired. She performed better on simple words than ambiguous words, reflecting a clear orthographic ambiguity effect (simple words: 31/50, 60%; mildly ambiguous words: 12/50, 24%; *X*^2^ (1) with continuity correction = 13.21, *p* < 0.001). In fact, CHS's performance decreased sharply as the degree of ambiguity increased. The more orthographically ambiguous a word was, the worse she performed when spelling it.

A total of 350 errors were recorded because CHS often made more than one error in a single word, particularly in ambiguous words. Furthermore, she never wrote a word without first repeating it and then spelling out each letter. Some words, however, she wrote immediately and correctly or with errors that preserved their whole form. CHS tended to write what she heard and to remove all types of orthographic ambiguity from the word stimuli. For example, when she was not sure if there was a ta marbu:ṭa in the word, she placed the word in another context and loudly pronounced the ta marbu:ṭa to check whether it could exist in that context; after this, she wrote the word.

CHS mainly produced visual (8/19, 42%) and phonologically nonplausible errors, including omission, substitution, and metathesis (9/19, 47.36%) in spelling unambiguous words. The remaining two errors consisted of one phonologically plausible error and one morphological error. As shown in Table 5, CHS mostly produced phonologically nonplausible errors (110/132, 83.3%) on ambiguous words. Most of these errors were made in contexts that are ambiguous in the Arabic language, namely, long vowels, ta marbu:ṭa, and the grapheme ʔ. CHS only spelled 46.3% (81/175) of long vowels correctly and mainly tended to omit them (e.g., غاز/ga:z/ (gas)→غز/gaz/). She spelled the ta marbu:ṭa correctly in 39.13% (18/46) of occurrences. Its omission (e.g., مساحة/misa:ha/(space)→مساح/misa:h/) was also predominant in CHS's errors.

Finally, CHS also exhibited notable difficulty spelling the ambiguous grapheme ʔ, which has seven allographs (26/82, 31.7%). Again, she often tended to omit it (e.g., مئزر/miʔzar/ (apron)→مزر/mizar/) (42/56, 75%). In 14 of which CHS omitted the Hamza but preserved the adjacent letter (its seat or support), such as مازق/maziq/for مأزق/maʔziq/ (trouble), تيو/tayu:/, for تنبؤ/tanabuʔ/ (prediction) and/ري riya/ for رئة/riʔa/ (lung), these errors reflect a simplification of the bigram/ʔ/into one letter (أ→ا, ؤ→و, and ئ→ى).

In a few cases, she substituted another allograph for the Hamza (11/56, 19.64%; e.g., بئر/biʔr/ (a well)→بءر/biʔr/) or substituted a visually similar letter (3/56, 5.35% e.g., فائزة/faʔiza/ (a feminine winner)→فايز/fayz/). Interestingly, CHS made more errors in writing the Hamza (37/43, 86%) in the middle position of the word than in the initial (8/21, 38%) and final (11/18, 21.9%) positions. This can be explained by the fact that the spelling of the Hamza is less ambiguous in the initial position (where it is always written with the letter ا) and the final position (no support of a letter but written on the line after a long vowel) than in the middle position. Finally, to ensure that the ambiguity effect was not simply due to word length, we compared CHS's performance on simple and ambiguous words of same letter length. For stimuli of 3 letters, CHS correctly spelled 18 out of the 28 simple words (64.3%) and only 4 out of the 15 ambiguous words (26.7%) (*p* = 0.01); for stimuli of 4 letters, CHS correctly spelled 13 out of the 22 simple words (59.1%) and only 10 out of the 51 ambiguous words (19.6%) (*p* = 0.001). This comparison could not be made for words of more than 4 letters since the letter length of simple words was limited to 4. Nevertheless, this analysis rules out, at least partially, the fact that CHS spelling difficulties were linked to the length of words rather than to their ambiguity level.

As shown in Table 4, CHS's performance on the writing-to-dictation of nonwords was also substantially impaired. On this task, she produced lexicalizations, letter substitutions, omission of long vowels, and no response. However, her performance was much better on simple than ambiguous nonwords (*X*^2^ (1) with continuity correction = 7.3, *p* < 0.01). Letter substitution errors were most common on simple nonwords (66.7%), while lexicalizations occurred chiefly on ambiguous nonwords (83.3%).

## 4. Discussion

The aim of this study was to investigate the influence of the ambiguity of the Arabic graphic system on CHS' spelling abilities. She presented with severe mixed agraphia characterized by impaired access to orthographic representations in the output lexicon and by impairment of the sublexical route. She also showed greater difficulty writing ambiguous than simple words, suggesting reliance on the non-lexical route of spelling. The results of the experimental study revealed that orthographic ambiguity had a clear influence on CHS's ability to spell both words and nonwords. Her errors' pattern suggested that the ambiguity effect is linked with the orthotactic level of spelling. An additional constraint for the writer directly derives from the particularities of the Arabic graphic system: ta marbu:ta is a silent letter; short vowels are not represented in writing; letters representing long vowels can also be consonants).

In fact, CHS had substantial difficulties with orthographic ambiguity and tended to omit the ambiguous graphemes, resulting in phonologically plausible errors. Some of these errors (omission of long vowels and the ta marbu:ṭa and Hamza misspellings) are very similar to those observed in children learning to write Arabic [10, 12] and suggest more reliance on the sublexical route in the face of lexical ambiguities than is observed in skillful writers. However, unlike CHS, young learners do not make errors reflecting the adoption of the lexical-semantic route, such as the preservation of the whole word form and misplacement of subtle details. Therefore, CHS's performance suggests that both routes of spelling are partially impaired, allowing her to rely on both to a certain degree.

CHS's pattern of impairment is consistent with the *summation hypothesis*, which suggests that the lexical-semantic and the sublexical routes interactively contribute to spelling [23, 24]. Support for this hypothesis comes from studies of healthy [25, 26] and neurologically impaired participants [4, 27]. For example, in healthy participants, previously heard rhyming words have been shown to influence the spelling of nonwords (e.g., the nonword /vi:m/ was spelled VEME following the priming word “theme” and VEAM when preceded by the priming word “dream”) [26]. In neuropsychological studies of individuals with agraphia, support for the *summation hypothesis* is mainly based on qualitative analyses of spelling errors. For example, Rapp et al. [4] demonstrated the interaction of the lexical-semantic and sublexical spelling routes in LAT, an individual with acquired agraphia who produced phonologically plausible errors such as *knolige* instead of *knowledge*. These errors involved low-frequency phoneme-to-grapheme correspondences (e.g., the segment KN), which are unlikely to result from the sublexical route, but rather suggest the involvement of the lexical-semantic route.

In CHS's case, support for the *summation hypothesis* also comes from a qualitative analysis of her spelling errors. Due to the peculiarities of the Arabic graphic system, many of these errors reflect a combination of the lexical-semantic and sublexical processes of spelling. In Arabic, the strict application of phoneme-to-grapheme conversion rules may lead to the omission of long vowels, the ta marbu:ṭa, and the grapheme ʔ. The decision to write or not write these graphemes is essentially based on the lexical-semantic route of spelling. CHS's strong tendency to omit these graphemes strongly suggests that she resorted to the sublexical route of spelling. However, her tendency to preserve the global shapes of words, along with overlooking details that are poorly represented graphically, such as dots and small symbols, suggests the involvement of the lexical-semantic route of spelling.

Finally, like LAT, the individual reported by Rapp et al. [4], CHS also produced errors that suggest the interaction of the two spelling routes. For example, she wrote the word برميل/bir mi:l/ (barrel) as وميل/wa mi:l (nonword)/, producing errors on the first two graphemes but correctly spelling the second word segment; although, it includes an ambiguous long vowel. In this error and others, simplification of the first syllable structure (CVC to CV) and confusion of the consonants /b/and/w/ suggest the intervention of the sublexical route of spelling. Another example is the production of قفزا instead of قفاز for /qufa:z/ (gloves). In this error, CHS first omitted the long vowel a: but still had the lexical knowledge that the word included this grapheme; so, she placed it at the end of the word.

Overall, CHS produced few semantic errors in written spelling. This pattern of performance also could reflect the integration of the two spelling routes. Like CHS, RCM, the individual with acquired agraphia reported by Hillis, Rapp, and Caramazza [28], had a postsemantic deficit that affected access to the orthographic output lexicon. RCM first produced semantic errors in spelling, but, over the course of recovery, these errors declined in number as RCM's sublexical spelling abilities improved. According to the authors, information recovered via the sublexical route helped prevent semantic errors resulting from the deficit in the activation of lexical representations. We also suggest that the partial preservation of the sublexical route in CHS might explain the small proportion of semantic errors she produced when spelling.

The pattern of performance observed in CHS can be explained by the two-route connectionist model of spelling proposed by Rapp et al. [4]. In this model, the information in the lexical-semantic route flows unidirectionally from the semantic system to the lexical orthographic representations. This information then flows bidirectionally between these representations and the graphemic layer. The sublexical route is implemented in a phoneme-to-grapheme conversion layer whose output also feeds the graphemic layer. In spelling-to-dictation, the two routes are activated simultaneously, and the interaction of their outputs then allows the production of the letter string. Although both routes were partially affected in CHS, the interaction of the two spelling routes can explain the types of errors she produced: phonologically plausible errors are explained by the application of phoneme-to-grapheme conversion rules in the sublexical route, and her tendency to preserve the global shapes of words suggests the involvement of the lexical-semantic route.

In a previous study, we demonstrated that CHS's reading skills were also impaired and characterized by a profile typical of deep dyslexia, along with letter-by-letter reading [19]. This profile was attributed to her brain damage, which is localized in the posterior cerebral artery territory and the left occipital region, two regions, respectively, associated with deep dyslexia [29] and letter-by-letter reading [30]. In written spelling, we have shown that, although CHS's performance met some of the criteria for deep agraphia, such as the lexicality effect (i.e., better performance in spelling words than nonwords), she produced very few semantic and morphological errors. Moreover, she also exhibited an orthographic ambiguity effect that cannot be easily explained in the context of deep agraphia. Béland and Mimouni [31] also reported deep dyslexia in an Arabic-speaking individual who suffered a stroke that affected the left peri-Sylvian area. However, to the best of our knowledge, the present study is the first to specifically explore acquired spelling impairment in an Arabic-speaking individual. The left hemisphere peri-Sylvian network has been shown to be involved in spelling and phonological processing skills in healthy subjects [32]. When lesioned, as in CHS, this network is associated with impairment of the sublexical and lexical-semantic routes of spelling [33].

Phoneme-to-grapheme correspondences are relatively regular in Arabic. However, this language has peculiarities that pose challenges to schoolchildren and to individuals with acquired deficits in written language, such as CHS [34]. However, from a crosslinguistic and qualitative point of view, the errors she produced on spelling tasks are directly linked to the ambiguity of the Arabic graphic system and more particularly to the orthotactic level of processing of long vowels, the phoneme/ʔ/, and the ta marbu:ṭa .

CHS spelling accuracy was not affected by lexical factors such as word frequency, imageability, and grammatical class. The error types observed in CHS (i.e., phonologically nonplausible errors: substitution, omission, inversion, and addition of graphemes) were relatively similar for words and for nonwords, as well as in oral and written spelling-to-dictation tasks. All these features are characteristics of a deficit to the graphemic output buffer (GOB) [2]. This deficit can be found in isolation [35] or in association with deep agraphia [36]. Based on the production of letter position errors in writing in AE, a Hebrew-speaking individual was also attributed to a selective deficit to the GOB [17]. However, unlike CHS, AE showed no characteristics of deep agraphia, and his performance was not influenced by orthographic consistency. In CHS, the deficit to the GOB co-occurred with an orthographic ambiguity effect. The qualitative analysis of her errors showed that letter substitutions were more frequent in unambiguous words, whereas letter omissions were more frequent in ambiguous words. In 1998, Annoni et al. described a case of selective deficit to the GOB in which an irregularity effect was observed, as in CHS. According to the authors, an orthographic regularity effect may occur at a postlexical stage by adding additional load to the processes of maintaining graphemes at the graphemic planning stage. This hypothesis was however not supported by additional studies and should therefore be considered with caution.

The errors CHS produced on spelling tasks are directly linked to the ambiguity of the Arabic graphic system. Since it is essentially consonantal, the mental representation of vowels is weaker than that of consonants, leading to greater concentration of attentional resources on consonants at the expense of vowels. From across-linguistic perspective, this finding indicates that the structure of the Arabic GOB differs from the western graphemic buffers in which the consonants and the vowels are equally represented.

This study has the usual strengths and weaknesses of single-case studies. This in-depth analysis of CHS's performance allowed us to challenge theoretical models of spelling. However, our findings cannot be generalized to all cases of agraphia in Arabic speakers. Further studies are needed to confirm the influence of Arabic orthographic features on spelling disorders. Further studies should also explore the potential relationship between agraphia in Arabic and deficits affecting verbal working memory and visuospatial processing. Moreover, a crosslinguistic assessment of writing in both Arabic-French would have been very interesting to confirm the potential overlapping between these two scripts in CHS. This is also a limitation in this single-case study. From a clinical standpoint, CHS's spelling profile could be useful for the diagnosis of acquired spelling disorders in Arabic-speaking individuals and could help guide clinicians to design credible tools for the assessment and treatment of such patients.

## Figures and Tables

**Table 1:** Performance of CHS and control participants on neuropsychological tests.

Cognitive domain	CHS	Norms and control data
*General cognition*		
MMSE (Arabic version)	24^∗^	26
*Short-term memory*		
Digit span forward-WAIS	2^∗^	ONR
*Constructional abilities*		
Copy of Rey-Osterrieth figure (/36)	21.5^∗^	*Z* = −3.07
*Visuoperceptual functions*		
Length match task–BORB (30)	17 (56.7%)^∗^	*Z* = −2.81
Object decision easy-BORB (32)	26 (81.25%)^∗^	*Z* = −3.21
Object decision hard-BORB (32)	19 (59.4%)^∗^	*Z* = −3.64
*Executive functions*		
Trail making test		
(i) Part A-time (sec)	103^∗^	*Z* = −7.38
(ii) Part B–time (sec)	333^∗^	*Z* = −16.73
Digit span backward-WAIS	0^∗^	ONR
Hayling test		
(i) Automatic condition (number of correct responses)	14	*Z* = −1
(ii) Inhibition condition (number of correct responses)	1^∗^	*Z* = −3.66
*Episodic memory*		
Immediate recall of Rey-Osterrieth figure (/36)	5.5^∗^	*Z* = −2.5

^∗^ indicates a score below the norm, expressed in *Z* score; ONR: outside normal range; MMSE: Mini-Mental State Examination; WAIS: Wechsler Adult Intelligence Scale III; BORB: Birmingham Object Recognition Battery.

**Table 2:** Performance of CHS and control participants, mean and (S.D.), on language tests.

Language domain	CHS	Controls	Modified *t*-test/chi^2^
*Verbal input analyses*			
Auditory discrimination (60)	53 (88.3%)^∗∗^	59.2 (1.1)	*t* (4) = −5.14; *p* = 0.006
Orthographic discrimination (78)	77 (98.8%)	77.6 (.89)	*t* (4) = −.61; *p* = 0.571
*Lexical access*			
Orthographic lexical decision (120)	69 (57.5%)^∗∗∗^	116.2 (1.5)	*t* (4) = −28.72; *p* < 0.001
(i) Words (60)	52 (86.7)^∗∗^	59.4 (1.3)	*t* (4) = −5.19; *p* = 0.006
(ii) Nonwords (60)	17 (28.3)^∗∗∗^	56.8 (1.9)	*t* (4) = −19.12; *p* < 0.001
Auditory lexical decision (120)	113 (94.16%)^∗^	118 (1)	*t* (4) = −4.56; *p* = 0.01
(i) Words (60)	59 (98.33%)	60 (0)	*X* ^2^ (1) = 00; *p* = 1.000
(ii) Nonwords (60)	54 (90%)^∗^	58 (1)	*t* (4) = −3.65; *p* = 0.02
*Phonological processing*			
Repetition			
(i) Words (72)	72 (100%)	72 (00)	—
(ii) Nonwords (40)	19 (47.5%)^∗∗∗^	40 (00)	*X* ^2^ (1) = 25.82; *p* < 0.001
Auditory rhyming judgment task (64)	43 (67.18%)^∗^	59.82 (4.14)	*t* (4) = −3.7; *p* = 0.02
Written rhyming judgment task (64)	31 (48.43%)^∗^	54.29 (5.78)	*t* (4) = −3.67; *p* = 0.02
*Semantic processing*			
Word-to-picture matching			
(i) Auditory modality (30)	30 (100%)	30 (00)	—
(ii) Written modality (30)	21 (70%)^∗∗^	30 (00)	*X* ^2^ (1) = 8.36; *p* = 0.004
Semantic similarity judgment task			
(i) Auditory modality (40)	36 (90%)^∗^	39.37 (.95)	*t*(4) = −3.23; *p* = 0.03
(ii) Written modality (40)	33 (82.5%)^∗∗^	38.55 (1.23)	*t* (4) = −4.11; *p* = 0.01
*Spoken production*			
Spoken picture naming (76)	52 (68.4)^∗∗∗^	74 (.70)	*t* (4) = −28.69; *p* < 0.001

^∗^ *p* < 0.05; ^∗∗^*p* < 0.01; ^∗∗∗^*p* < 0.001.

**Table 3:** Performance of CHS and control participants (mean and S.D.) on writing to dictation and written picture naming tasks.

Spelling tasks	CHS	Controls	Modified *t*-test/chi^2^
*Writing to dictation*			
*Letters and syllables*			
Letters (28)	23 (82.14%)	28 (00)	*X* ^2^ (1) = 3.51; *p* = 0.061
Syllables with long vowels (30)	13 (43.33%)^∗∗∗^	30 (00)	*X* ^2^ (1) = 14.14; *p* < 0.001
Syllables without long vowels (30)	19 (63.33%)^∗∗∗^	30 (00)	*X* ^2^ (1) = 14.14; *p* < 0.001
*Words*			
Low-frequency words (20)	4 (25%)^∗∗∗^	20 (0)	*X* ^2^ (1) = 23.43; *p* < 0.001
High-frequency words (20)	6 (30%)^∗∗∗^	20 (0)	*X* ^2^ (1) = 18.57; *p* < 0.001
Low-imageability words (30)	10 (33.33%)^∗∗∗^	29.8 (.4)	*t* (3) = −45.18; *p* < 0.001
High-imageability words (30)	6 (20%)^∗∗∗^	30 (0)	*X* ^2^ (1) = 36.73; *p* < 0.001
Closed-class words (20)	4 (20%)^∗∗∗^	20 (0)	*X* ^2^ (1) = 23.43; *p* < 0.001
Open-class (20)	5 (25%)^∗∗∗^	20 (0)	*X* ^2^ (1) = 20.90; *p* < 0.001
*Nonwords* (19)	3 (16%)	19 (0)	*X* ^2^ (1) = 24.29; *p* < 0.001
*Oral vs. written spelling of words*			
Oral spelling (72)	14 (19.4%)^∗∗∗^	71.66 (.57)	*t* (3) = −87.51; *p* < 0.001
Written spelling (72)	21 (29.2)%^∗∗∗^	72 (0)	*X* ^2^ (1) = 75.9; *p* < 0.001
*Written picture naming*			
Object pictures (55)	23 (41.8%)^∗∗∗^	53.2 (1.09)	*t* (3) = −25.29; *p* < 0.001

^∗∗∗^ *p* < .001. Note: the database Aralex [22] was used to control word frequency. There exists no data for the degree of the imageability of Arabic words. Therefore, 19 participants (mean age: 37.7 years; age range: 21-52 years; education level: <12) were asked to rate the words for imageability (i.e., their ability to evoke mental imagery) on a 7-point Likert scale (1 = very low imageability; 7 = very high imageability). The words that participants scored 1-3 were considered low-imageability words, while those scored 5-7 were considered high-imageability words. The mean rating was 2.51 (SD = 0.61) for low-imageability words and 5.95 (SD =1.13) for high-imageability words, and the difference between the two types of word was significant (*X*^2^ = 113.89, *p* = 0.0001).

**Table 4:** Performance of CHS and control participants (mean and S.D.) on the spelling-to-dictation task of ambiguous and unambiguous words and nonwords.

Stimulus types	CHS	Controls	Modified *t*-test/chi^2^
*Words*			
(1) Unambiguous words (50)	31(62%)	50 (0)	*X* ^2^ (1) = .21.05, *p* < 0.001
(2) Mildly ambiguous words (50)	12 (24%)	50 (0)	*X* ^2^ (1) = 58.1, *p* < 0.001
(3) Ambiguous words (50)	4 (8%)	49 (.7)	*t*(5) = −58.68; *p* < 0.001
(4) Very ambiguous words (50)	2 (4%)	49.2 (.83)	*t*(5) = −51.91; *p* < 0.001
Total score (200)	49 (24.5%)	198.2 (.83)	*t*(5) = −164.09; *p* < 0.001
*Nonwords*			
(1) Unambiguous nonwords (20)	11 (55%)	18.6 (.89)	*t*(5) = −7.79; *p* < 0.001
(2) Ambiguous nonwords (20)	2 (10%)	19.4 (.54)	*t*(5) = −29.41; *p* < 0.001
Total score (40)	13 (32.5%)	38 (.7)	*t*(5) = −32.6; *p* < 0.001

**Table 5:** Distribution of error types produced by CHS on writing to dictation ambiguous words.

Graphemes	Error types				Total number of errors
	Substitution	Omission	Inversion	Addition	
Letters (consonants) (*n* = 603)	105 (66.46%)	36 (22.78%)	9 (5.69%)	8 (5.06%)	158
Long vowels (*n* = 175)	8 (7.69%)	81 (77.88%)	5 (4.81%)	10 (9.61%)	104
Ta marbu:ṭa (*n* = 46)	4 (12.5%)	24 (75%)	—	4 (12.5%)	32
Hamza (*n* = 82)	14 (25%)	42 (75%)	—	—	56

## Data Availability

Data are unavailable.

## Appendix

### A. Written Narration of the Bank Robbery Scene

- Written sample of CHS's written production

(b) Transcription of CHS's written production in Arabic alphabet

سَرق المور .....على للي يبكي .... في ...رجال (رجل).....شرطة .....(سارق الأموال) .يسرق .... الموال.....المومام ....

اموطنـ.... يهرب ...يهلب..... امسيس (لصوص)..... بنك .....سعدن (ساعدوني) .......شرطة . ......يتصل.....يتصلب

سرق. اموطن (المواطنون) مراة ييقم (المرأة ترتجف) شرق (سرق).....

(c) Transcription of CHS's written production in Latin alphabet

/saraqa al-mu:r….al-mu:ma:m…. al-muwa:l…..yasriq…… šurṭa…..ridʒa:l……fi:….. ʕala:…lli: ….yabki:…..yataṣalab……yataṣil…….. šurṭa……sʕdn…..bank….ʔamsi:s……

yahlub….yahrub….ʔmawṭin…. ʔmawṭin ..ʔmawṭi….. marʔa…..yayaqim….šaraq …saraq/

(d) English translation of CHS's written production

Money thief [misspelled] … he steals … Police … men … in … on …/lili:/… he cries … he “hardnes” [visually close to “call” in Arabic] … he calls … police … help me [misspelled] … Bank … Thieves [misspelled] …/yahlab/[visually close to “he runs”] … he runs/ʔ/[“citizens” misspelled] … [“the woman is shivering” misspelled] … stole [self-corrected]

### B. Examples of Error Types Produced by CHS in Spelling by Dictation

Unambiguous words

- Substitution: ،مقص←مقط (scissors)
- Omission:بلسم ← بلم (balm)
- Inversion:مكتب ← مكبة (desk)
- Phonologically plausible: مصنع ← مسنع (factory)
- Morphological errors مكتبة: (library)مكتب ← (desk)
- Errors preserving global word form: طفل←الطقل (child)

Ambiguous words

- Substitution: long vowel; طريق←طراق (road)/Hamza; سيالسؤال ← (question)
- Omission: long vowel; غاز←غز (gas)/Hamza;خط خطأ←(mistake)/ta marbu:ṭa; مساحة ← مساح (space)
- Inversion: long vowel;قاموس ← قاومس (dictionary)
- Phonologically plausible: صابون ← صبون (soap)
- Morphological errors: سيارات ← سيارة (cars)
- Semantic errors: رائب ← (milk) حليب
- Errors preserving global word form عنوان ← عيوان (address)

Nonwords

- Substitution: خزابه ← حزابه
- Omission: ذفذع ← ضدع
- Inversion: حسل←سحل
- Lexicalisation: طيبب ← طيب
